# Ovarian tissue vitrification is more efficient than slow freezing to preserve ovarian stem cells in CF-1 mice

**DOI:** 10.5935/1518-0557.20190057

**Published:** 2020

**Authors:** Paula Barros Terraciano, Tuane Alves Garcez, Markus Berger, Isabel Durli, Cristiana Palma Kuhl, Vitória de Oliveira Batista, Raquel de Almeida Schneider, Jaquelline Festa, Emily Pilar, Marcela Goulart, Charles Ferreira, Eduardo Pandolfi Passos, Elizabeth Cirne Lima

**Affiliations:** 1 Centro de Pesquisa Experimental, Laboratório de Embriologia e Diferenciação Celular, Hospital de Clínicas de Porto Alegre, Brazil; 2 Programa de Pós Graduação em Ciências da Saúde: Ginecologia e Obstetrícia, Universidade Federal do Rio Grande do Sul, Brazil; 3 Departamento de Patologia Clínica, Faculdade de Veterinária, Universidade Federal do Rio Grande do Sul, Brazil; 4 Centro de Pesquisa Experimental, Unidade de Patologia Experimental, Hospital de Clínicas de Porto Alegre, Brazil

**Keywords:** ovary, vitrification, stem cell

## Abstract

**Objective:**

The aim of this study was to investigate the efficacy of protocols for mice ovary cryopreservation to compare the differences in Mouse Vasa Homologue expression (a germline cell marker) and ovarian viability after vitrification or slow freezing.

**Methods:**

Female CF1 mice aged 40-45 days were randomly divided into three groups: Control, vitrification or slow freezing. Their ovaries were surgically removed, rinsed in saline solution and cryopreserved. For vitrification, we used a commercial protocol and for slow freeze, we used 1.5 M ethylene glycol (EG) as cryoprotectant. After that, the ovaries were processed for histological an immunohistochemical analysis, and counting of primordial, primary, pre-antral and antral follicles.

**Results:**

No significant difference was found in the proportion of high-quality primordial, primary and pre-antral follicles after thawing/warming in the slow freezing and vitrification groups. The immunohistochemistry for MVH antibody demonstrated that the slow freeze group had a higher number of unmarked cells (*p*=0.012), indicating a harmful effect on the MVH expression in the ovarian tissue, where the cell structure is complex.

**Conclusion:**

Although both protocols indicated similar results in the histological analysis of follicular counts, the vitrification protocol was significantly better to preserve ovarian stem cells, an immature germ cell population. These cells are able to self-renew having regeneration potential, and may be effective for the treatment of ovarian failure and consequently infertility.

## INTRODUCTION

Ovarian transplantation has been used for many years in animal models to study ovarian endocrine function (Bagwell *et al*., 1976), and it was later adapted for ovarian function studies after cryopreservation (Sugimoto *et al*., 2000). Ovarian cryopreservation research is performed for the purpose of strain preservation (Dorsch *et al*., 2004) and to optimize the procedure for use in human female fertility preservation programs (Demeestere *et al*., 2009). Fertility preservation in human female aims to preserve/restore fertility in girls and young adult women planned to undergo potentially gonadotoxic cancer therapy (Meirow & Nugent, 2001; Bastings *et al*., 2016; Donnez *et al*., 2011). However, only a very limited number of live births have been reported and it is most likely that a large number of transplantation attempts have been performed; the procedure still needs further development to increase its effectiveness. Factors that should be improved are cryopreservation protocols and surgical transplantation procedures (Kagawa *et al*., 2009; Sheikhi *et al*., 2011; Ackermann *et al*., 2017).

Most of the follicular loss in cryopreserved tissue does not occur during the cryopreservation/thawing processes, but rather during the warm ischemic time after retransplantation (Liu *et al*., 2002; Gosden, 2000). Interventions such as transplantation to granulation tissue (Israely *et al*., 2006) or incubation of the pretransplantation tissue with growth factors (Schnorr *et al*., 2002), vitamin E (Nugent *et al*., 1998), or other antioxidants (Weissman *et al*., 1999; Kim *et al*., 2004; Sapmaz *et al*., 2003) have shown moderate or no effect to increase follicular survival. Whole ovary transplantation has been suggested as an approach to overcome the deleterious effects of prolonged ischemic time after the tissue reintroduction (Jadoul *et al*., 2007; Martinez-Madrid *et al*., 2007; Bedaiwy & Falcone, 2010; Bromer & Patrizio, 2009). Thus, alternative techniques for whole ovary cryopreservation and transplantation with vascular anastomosis should be stimulated (Silber *et al*., 2008; Imhof *et al*., 2006; Wang *et al*., 2002). Indeed, live births have been demonstrated after whole ovary cryopreservation and vascular retransplantation, both in sheep (Imhof *et al*., 2006) and in rat models (Silber *et al*., 2008), although the procedure as a whole has low effectiveness, leading to low live-birth rates.

Considering the difficulties of ovarian tissue transplantation techniques, improvements in cryopreservation procedures are needed. Therefore, the aim of this study was to investigate the efficacy of protocols for cryopreservation of mice ovary, and compare the differences in Mouse Vasa Homologue expression (a germline cell marker) and ovarian viability after vitrification or slow freezing.

## MATERIALS AND METHODS

### Animals

Female CF-1 mice, aged 28 to 30 days with average weight of 29.29±2.9g, were used. The animals were kept in group cages under controlled conditions (23ºC and 12-hour light/dark cycles). They were fed pelleted food and tap water *ad libitum*. After an acclimatization period, the mice were randomly divided into three groups and were submitted to vaginal cytology, for confirmation of the estrous cycle before euthanize, with isoflurane overdoses, to remove the ovaries for experiments. The Animal Ethics Commission of HCPA (CEUA-HCPA 16-0169) approved these experiments.

### Experimental Groups

The experiment was designed to compare the viability and MVH expression by ovarian cells after different cryopreservation processes. Each group consisted of ten animals (n=20 ovaries) randomly allocated to the following groups: fresh control ovaries (C) or ovaries cryopreserved by either vitrification (VIT) or slow-freezing (SF).

### Ovaries

The ovaries were collected after slaughtering the animals by anesthetic overdose with isoflurane (5-10% at 100% O_2_), and they were dissected to remove adipose and mesenteric tissue. Immediately after, the ovaries were submitted to cryopreservation according with their experimental group. The right and left ovaries of the same animal were separately cryopreserved for different analysis.

### Cryopreservation

Whole ovaries subjected to slow freezing (SF) were incubated for 15min in DMEM supplemented with 1.5M Ethylene Glycol (EG) and 0.5 M sucrose at 4ºC, added to 1.5mL cryovials containing 1.5M EG solution (0.5mL) and placed into a container at room temperature (Cryo Freezing Container; Nalgene) with isopropanol. The container was placed in a -80ºC freezer for 24 hours to allow freezing at a rate of approximately 1ºC/minute, and then it was placed into liquid nitrogen and stored until thawing. The ovaries of the vitrification group (VIT) were processed with a commercial kit (Vit Kit^®^ - Freeze, Irvine Scientific, California). The ovaries were immersed in the equilibrium solution containing 7.5% ethylene glycol (EG) and 7.5% dimethyl sulfoxide (DMSO) in DPBS supplemented with 20% FBS for 10 min at room temperature, and then transferred to a vitrification solution (15% EG, 15% DMSO and 0.5M sucrose) for 2 min, and were then placed on a piece of sterile gauze to remove excess medium and were then immediately frozen in liquid nitrogen (1.5mL cryovials).

### Thawing

After 30 days of storage, the cryovials were removed from the cryotank and placed into a water bath at 37ºC for 2 to 3 min to allow complete thawing. Slow-Freeze ovaries was rinsed in DMEM medium supplemented with decreasing concentrations of sucrose (0.5, 0.25, 0.1M) for approximately 5 minutes in each washing step. After these steps to wash out the cryoprotectant, the ovary samples were processed for further viability tests. The ovaries submitted to the vitrification process were quickly removed from the liquid nitrogen and fully immersed in the 37ºC water bath for 3 seconds. The ovary was placed directly into the thawing solution (1 M sucrose, 20% DSS and Gentamicin in M-199 medium) and incubated for 1 min. After that it was transferred to a dilution solution (0.5 M sucrose, 20% DSS, Gentamicin in M-199 medium), incubated for 4 min and finally transferred to the rinsing solution (20% DSS, Gentamicin, in M-199 Medium) and incubated for 4 min.

### Histological Analysis

The ovary tissues were removed, fixed in formalin, embedded in paraffin and stained with hematoxylin and eosin.

### Immunohistochemical Analysis

The germline MVH/DDX4 stem cell markers' expression was analyzed in ovary samples by immunohistochemistry. Briefly, the sections were incubated overnight at 4ºC with a polyclonal rabbit antibody anti-DDX4 (Abcam, Cambridge) at a dilution of 1: 200. After the incubation interval, the sections were rinsed and incubated with the goat anti-rabbit IgG (H + L) HRP (Millipore, Massachusetts) detection system at a dilution of 1: 200. The reaction was finally developed with Liquid DAB (Dako, California), according to the manufacturer's recommendations.

### Estrous cycle detection

The females were submitted to vaginal cytological analysis before euthanasia, as described by Ceschin *et al*. (2004), in order to confirm the estrous cycle stage. Vaginal suspensions were collected in 0.5% NaCl (0.25mL), and the smears were evaluated according to Cooper *et al*. (1998).

### Follicular Classification

The follicles were classified according to the modified criteria published by Oktay *et al*. (1998) as follows: the follicles were analyzed and categorized as primordial, primary, pre antral and antral. Primordial follicles were identified as normal, even when they had cytoplasmic and/or irregular contour vacuolization, since such characteristics were considered reversible.

### Statistics

The data was expressed as medians, percentages, quartiles, and ranges. Statistical comparisons were performed using the One-Way ANOVA for parametric variables, *p*<.05 was considered statistically significant. Statistical evaluations were performed using the PASW 18.0 software.

## RESULTS

### Estrus cycle and body weight analysis

**Figure 1:**
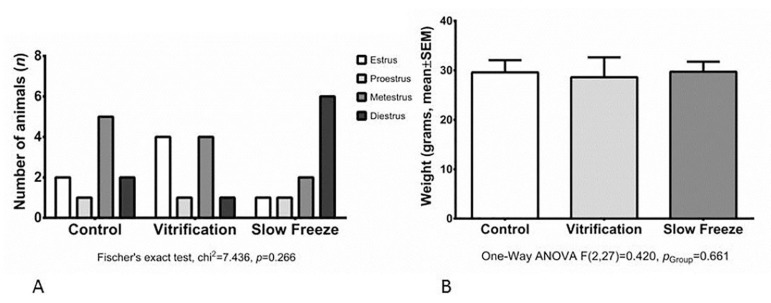
A. No statistical difference in the estrus cycle phase among the groups (*p*=0.266). B. No statistical difference in body weight among the groups (*p*=0.661)

The estrus cycle phase and body weight (29.29±2.9g) were evaluated and presented similar results (Figure 1).

### Histological evaluation

**Figure 2:**
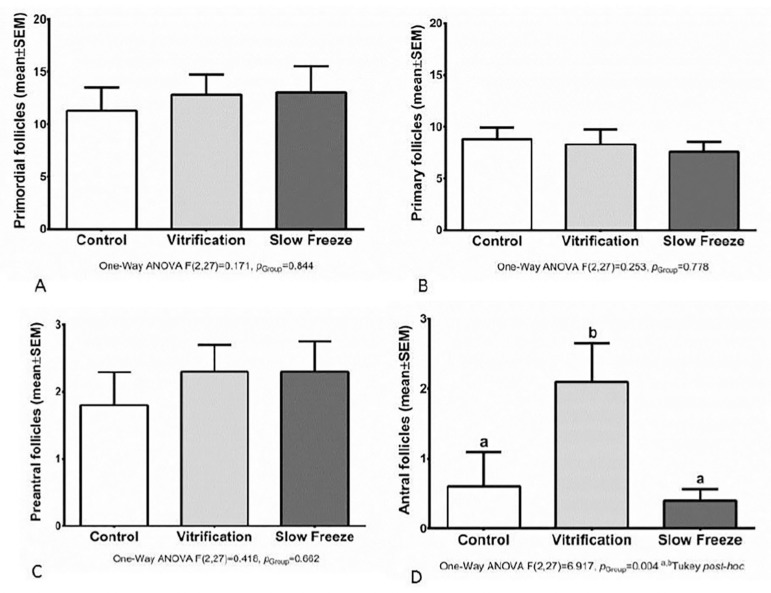
Number of follicles: primordial (A), primary (B), preantral (C) and antral (D) quantified from the hematoxylin and eosin technique. ab. Different letters indicate statistical difference

Hematoxylin and eosin slides were prepared to evaluate the presence of primordial, primary, pre antral and antral follicles. When the samples were submitted to slow freezing or vitrification processes a similar morphology was found among primordial, primary and pre antral follicles after the thawing/warming process. On the other hand when antral follicles were evaluated, we detected a significantly higher number of these structures on ovary samples submitted to the vitrification process (*p*=0.004) (Figure 2).

### Immunohistochemistry for MVH cells

**Figure 3:**
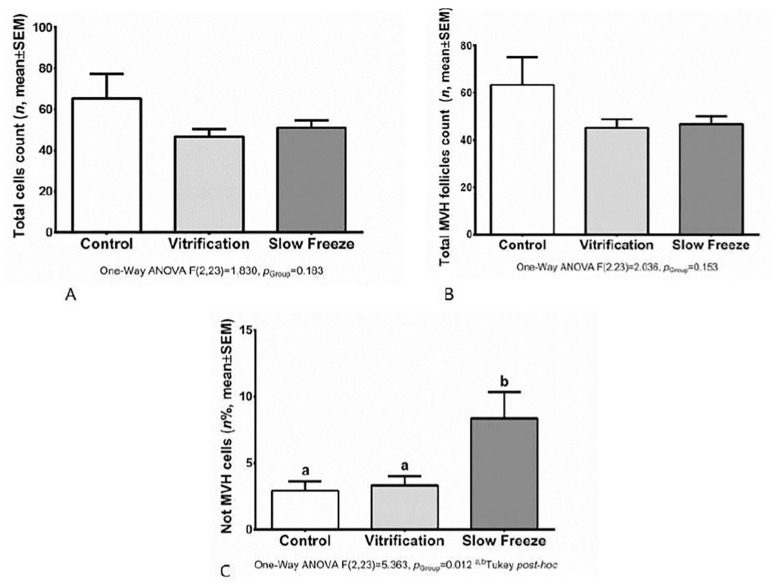
A: No statistical difference among groups in total cell count number (*p*=0.183). B: No statistical difference in total follicles count (*p*=0.153). C: Statistical difference among groups in percentage number of MVH+. Slow freeze group had a higher number of unstained cells (*p*=0.012)

**Figure 4:**
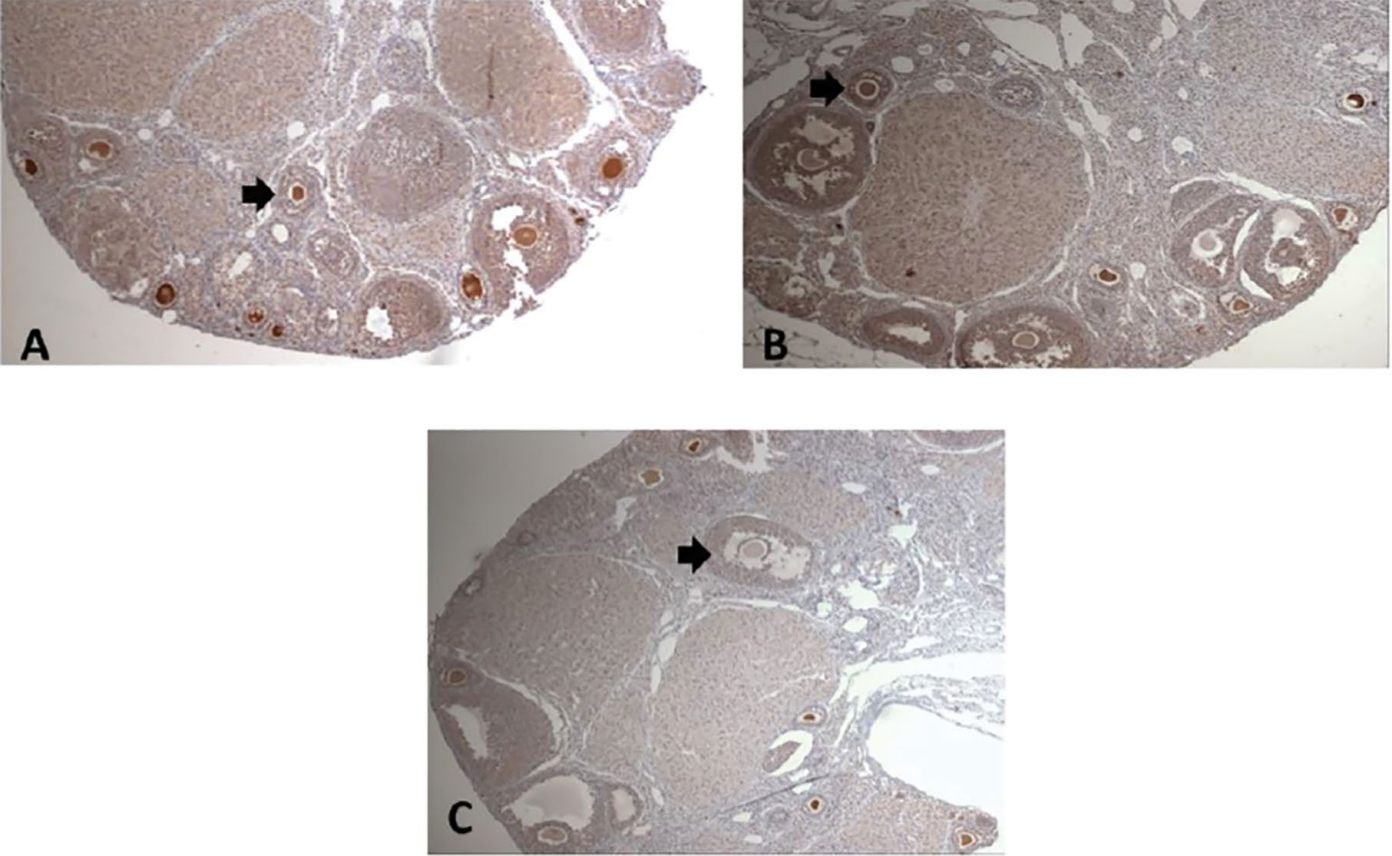
Immunohistochemical (IHC) sections from ovaries showing viable follicles. A: Control Group IHC for MVH+ Cells. B: Vitrification Group IHC for MVH+ Cells and C: Slow Freeze Group IHC for MVH+ Cells. Magnification 40x

The MVH expression in frozen/thawed ovaries was assessed by immunohistochemistry. The MVH+ and MVH- follicles were counted and compared with the total number of follicles found in each ovary sample from different groups (Figures 3 and 4). Total cells and total MVH positive cells were similar in different groups. When negative and positive MVH follicles where evaluated, we noticed a higher rate of negative MVH cells (no stained cells) on the ovaries submitted to the slow freeze process.

## DISCUSSION

Ovarian tissue cryopreservation is the primary treatment currently available to women at risk of losing their ovarian function due to cytotoxic therapy or radiotherapy. Its effectiveness is still fairly low. Around 15 live births have been reported (Bastings *et al*., 2016) since the introduction of this method, as an experimental procedure for more than 10 years (Oktay & Karlikaya, 2000), and the first healthy baby was born in 2004 (Donnez *et al*., 2004). Cell damage induced by ice crystals formation and how to optimize the ovarian transplantation procedure are major questions to address in order to increase the fertility rate after transplantation with cryopreserved ovaries.

Several animal models could be used to optimize cryopreservation protocols for human use. Sheep is the animal model that has been extensively used in ovarian cryopreservation research (Baird *et al.,* 1999; Almodin *et al*., 2004), considering ovine ovary size and because these animals have mono/diovulatory cycles. However, this experimental model is expensive when compared to rodent models and inconvenient to work with. A murine model has many advantages as an experimental model for research given that it is a small and inexpensive animal with high reproductive efficiency. Moreover, knowledge about ovarian function in mice, especially folliculogenesis, is well-reported (Faire *et al*., 2015). The rat ovary is nearly thirty-eight times smaller than human ovaries, the primordial follicle pool distribution within ovary structure is somewhat different, and these characteristics must be taken into consideration (Faire *et al*., 2015). DMSO has been widely used as cryoprotectant to preserve ovarian tissue, despite several others such as ethylene glycol, which has also shown effectiveness in ovarian fertility preservation procedures, being used in experimental and clinical applications (Lee *et al*., 2015).

The aim of this study was to compare two different cryopreservation protocols for mice ovaries in order to assess follicle viability and MVH expression after vitrification or non-automated slow freezing processes. Ovarian tissue cryopreservation efficiency is defined as the amount of viable primordial and primary ovarian follicles detected in the processed ovarian tissue, where these structures are potentially able to generate mature oocytes in adequate conditions.

In the present study, we compared the efficacy of two cryopreservation methods, applying a specific cryoprotectant solution to each of them. Vitrification was performed applying EG and DMSO (v/v), and the non-automated slow freezing process was performed with an EG containing the cryoprotectant solution.

Regarding the histological evaluation, no significant difference was found in the rate of viable primordial, primary and pre antral follicles in the control group (92.7%), after thawing/warming in the slow-freezing and vitrification groups, with 89.7% and 91.5% of viable follicles, respectively. On the other hand the number of antral follicles was significantly higher in ovary tissues submitted to the vitrification process (*p*=0.004) (Figure 2). This important result was probably due to the lower embryonic cell toxicity and higher cell membrane permeability of EG (Miyamoto & Ishibashi, 1978). Comparing four different cryoprotectant agents (Propanediol, Glycerol, DMSO, EG), Lucci *et al*. (2004) demonstrate more effective outcomes with DMSO- and PROH-based cryoprotectant agents, which preserved the structural integrity of somatic and germ cells. This difference can be explained due to interspecies peculiarities in ovarian tissue; probably bovine follicles are more sensitive to the EG toxic effects than other species. Furthermore, EG cryopreservation seems to be more effective (Candy *et al*., 1997) considering that it was possible to preserve 88% of morphologically normal follicles after thawing murine ovarian tissues; however, the authors warned that prolonged exposure to EG might decrease follicular viability. In another study with human ovarian tissue, 84% of follicles survived after cryopreservation in EG (Candy *et al*., 1997). These findings are in accordance with our results: EG demonstrated to be less toxic as a freezing solution, as shown by its superior preservation rates of ovarian tissue structural integrity. In addition, we found a significant number of antral follicles when ovary samples were cryopreserved by the vitrification process (*p*=0.04). In parallel, the group of experimental females was analyzed, and 80% of them were in the estrous phase, which corresponds to an ovulatory phase with increased follicular growth, or in metaestrus phase, which corresponds to the period exactly after ovulation, although there is no statistically significant difference among different analyzed cryopreservation ovary tissue processes when oestrus phases were compared.

In the last decade, several studies have yielded controversial results when comparing the conventional freezing process with vitrification (Newton *et al*., 1996; Keros *et al*., 2009; Oktem *et al*., 2011). Isachenko *et al*. (2009) tested vitrification versus the conventional freezing process of human ovarian tissue and concluded that conventional freezing is a better technique, since the preserved tissue conserves a higher development potential.

Based on the study by Zou *et al*. (2009), which was able to transplant ovarian stem cells into mice, and obtain a viable offspring, and Terraciano *et al*. (2014), which compared the ADSC or MVH cell transplant to restore fertility in mice with significant positive results, our initial purpose was to compare the MVH expression in different types of freezing processes for subsequent cell transplantation. In order to assess the MVH expression in frozen/thawed ovaries, we performed immunohistochemistry. MVH is the homologue of the Drosophila vasa gene, which is specifically expressed in all germ cell lineages and is known as a specific marker of reproductive cells (Encinas *et al*., 2012; Li *et al*., 2014). Therefore, in general, MVH is used as the marker of germ stem cells. In our study the MVH+ and MVH- follicles were counted and compared with the total number of follicles and we found a higher number of unstained cells in the slow freeze group (*p*=0.012). Our data showed that vitrification of ovarian tissue using combination of cryoprotectant agents (EG with DMSO) had no harmful effect on the morphology and MVH expression in the ovarian tissue, where the cell structure is complex.

The major difficulty with vitrification protocols is its high toxicity, due to the high concentration of cryoprotectants used, which can cause severe osmotic shock and compromise tissue survival after thawing (Rall & Fahy, 1985; Rall, 1987; Vajta *et al*., 1998). Toxicity reduction can be achieved using a combination of two cryoprotectants and a gradual exposure of the cells to the concentrated solutions prior to cooling. This technique was used in this study, similarly to other studies (Hasegawa *et al*., 2004; Liebermann *et al*., 2002). Although both protocols showed similar results in the histological analysis for follicular counts, the vitrification protocol was significantly better to preserve the ovarian stem cell population.

Ceschin *et al*. (2004) using IHC analysis with Ki-67 concluded that although both conventional freezing and vitrification were feasible methods for ovarian tissue cryopreservation, vitrification was associated with the recovery of a greater number of potentially viable primordial follicles in rats, similarly to ours conclusions.
